# Role of Suspended Particulate Matter for the Transport and Risks of Organic Micropollutant Mixtures in Rivers: A Comparison between Baseflow and High Discharge Conditions

**DOI:** 10.1021/acs.est.4c13378

**Published:** 2025-02-11

**Authors:** Lili Niu, Andrea A. E. Gärtner, Maria König, Martin Krauss, Stephanie Spahr, Beate I. Escher

**Affiliations:** † Department of Cell Toxicology, Helmholtz Centre for Environmental Research—UFZ, 04318 Leipzig, Germany; ‡ Key Laboratory of Pollution Exposure and Health Intervention of Zhejiang Province, Interdisciplinary Research Academy (IRA), Zhejiang Shuren University, 310015 Hangzhou, China; § Department of Exposure Science, Helmholtz Centre for Environmental Research—UFZ, 04318 Leipzig, Germany; ∥ Department of Ecohydrology and Biogeochemistry, Leibniz Institute of Freshwater Ecology and Inland Fisheries (IGB), Müggelseedamm 301, 12587 Berlin, Germany; ⊥ Department of Geosciences, Eberhard Karls University of Tübingen, Schnarrenbergstr. 94-96, 72076 Tübingen, Germany

**Keywords:** mixture risk, chemical analysis, bioassay, aquatic disturbance, contaminant redistribution

## Abstract

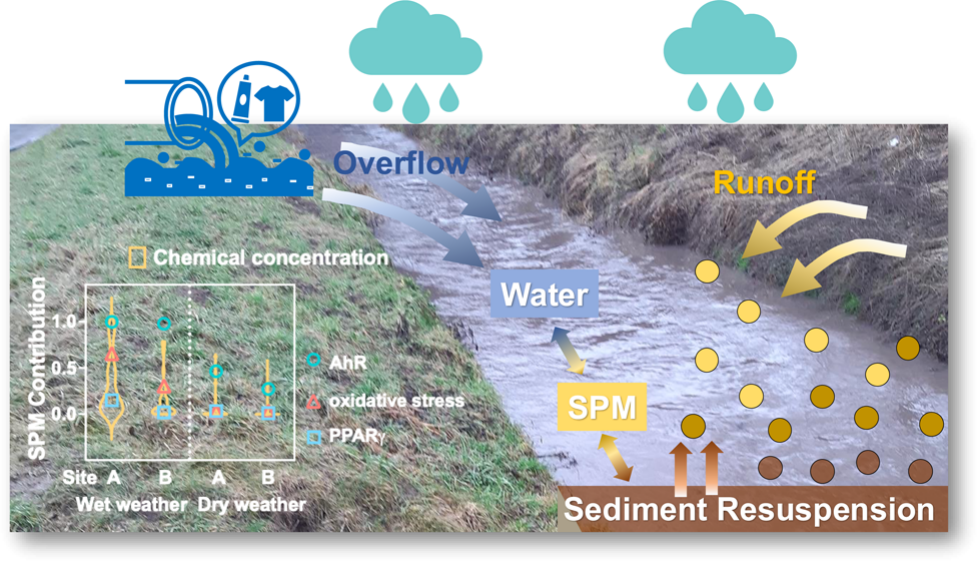

The partition dynamics of organic micropollutants between water and suspended particulate matter (SPM) in riverine ecosystems differs between dry and wet weather, as demonstrated at two sites at the Ammer River, Germany. One site was impacted by a wastewater treatment plant (WWTP) and the other by runoff of a mixed agricultural/urban area. Liquid and gas chromatography coupled to high-resolution mass spectrometry were used to quantify 415 organic chemicals, and their mixture effects were characterized with three in vitro bioassays indicative of the activation of the aryl hydrocarbon (AhR) and peroxisome proliferator-activated (PPARγ) receptors and the oxidative stress response. During wet weather, the total chemical concentrations and bioactivities in the water increased, but the concentrations in SPM did not change. As SPM levels increased, the SPM-bound chemicals contributed 6–16% to the overall concentrations in the water column during wet weather but only 0.1–0.9% during dry weather. The mixture effects were more strongly associated with SPM under wet conditions, particularly for AhR activity, where SPM accounted for over 90% of the observed effects. The AhR activity may therefore serve as an indicator for assessing the risks of SPM-related pollution in rivers. The high SPM-bound mixtures’ activation of AhR and oxidative stress response during rain were primarily caused by polycyclic aromatic hydrocarbons, indicating a major contribution of road runoff.

## Abstract 2

Chemical concentrations and mixture effects in extracts from rivers are dominated by suspended particulate matter during rain, but the dissolved phase dominates during dry weather.

## Introduction

Hydrologic characteristics regulate the behavior, bioavailability, and fate of organic micropollutants in aquatic environments.^1,2^ Suspended particulate matter (SPM) plays a vital role in the delivery and transportation of contaminants in rivers due to its mobilization from riverbeds or riverbanks and the sorbed micropollutants it carries.^3−6^ The SPM-associated chemicals can accumulate in bed sediments after deposition and resuspend to the water phase when the hydrodynamic conditions change. With the increased frequency and severity of storm events due to climate change, such resuspension–deposition cycles may occur more often. Heavy rainfall triggers various point and nonpoint sources of pollution to rivers, resulting in inputs of pollutants in particle-associated and dissolved form coming from agricultural runoff, road runoff, and sewer overflow.^7−10^ As a result, the near-equilibrium distribution of chemicals between the aqueous and particulate phases established under dry weather conditions would be disrupted, and a mobilization of organic micropollutants could occur.^11^ Müller et al.^12^ discovered that the SPM-bound fractions accounted for up to one-third of the total mass flux of organophosphates in rivers during storm events. Glaser et al.^13^ found linear correlations between SPM concentration and cytotoxicity as well as particle-associated organic contaminant loads. On the other hand, the river pollution may be diluted by increasing water flow and mixing with cleaner water. For example, lower concentrations of endocrine-disrupting chemicals in the water of the Lhasa River Basin in the Tibetan Plateau, China,^14^ and the Taihu Lake Basin, China,^1^ were found during the wet season due to greater dilution by increased precipitation and water flow compared to the dry season. In addition, the frequent precipitation and high water flow alleviate the affinity of corticosteroids to SPM, along with the noncontaminated natural particle inputs from soil erosion, resulting in lower chemical loadings in SPM in the Pearl River, China, during the wet season.^15^

In a previous study, we found that even under dry weather conditions with SPM concentration as low as 1 mg/L, the SPM-bound chemicals still accounted for up to 46% of the effect burden in the Ammer River, Germany, making SPM an important source of water contamination.^16^ However, it is still unclear whether an enrichment or dilution of river pollution dominates when a catchment is disturbed by rainfall.^17^ In order to compare the distribution patterns of contaminants between phases and the role of SPM under different hydrodynamic conditions, the same types of samples as previously studied in dry weather were collected during wet weather at the same sites. Target chemical screening and in vitro bioassays were used in a complementary manner to identify priority pollutants as well as the overall chemical burden and mixture effects. This integrated strategy has been widely applied to water bodies since it can not only estimate the occurrence of individual chemicals but also explain how much of the effects can be described by detected chemicals with the aid of iceberg modeling.^7,16^ More than 600 chemicals with a wide range of octanol/water partition coefficients (log *K*_ow_ of −0.47–8.68) belonging to diverse compound classes were included in the target analytical method, but only 415 chemicals overlapping with those detected in dry weather^16^ were quantified. Three environmentally relevant modes of action covering different stages of the cellular toxicity pathway, that is, the activation of aryl hydrocarbon receptor activity (AhR), the binding to the peroxisome proliferator-activated receptor γ (PPARγ), and the activation of the oxidative stress response, were used in the bioassay battery.

We hypothesize that rain events trigger a redistribution of chemical loads and mixture effects between phases, with SPM becoming a more important contributor to river pollution compared to dry weather conditions, although to varying degrees for different pollutant classes. In consequence, the mixture risk drivers might be altered due to the additional input sources during rain events. The main goals of this study were to (1) characterize the chemical and toxicological profiles of organic micropollutants in the water and SPM, (2) quantify the contribution of SPM-bound contaminants to water pollution, and (3) identify the primary mixture risk drivers in the river under high discharge conditions during wet weather, and (4) compare with observations under baseflow conditions during dry weather published previously.^16^ We did not focus on exceptional storm events, which are often observed during the summer, but rather on long periods of rain in the winter in the temperate climate of Central Europe. We differentiated between the chemicals bound to dissolved organic carbon (DOC) in the water and particulate OC by combining solid phase extraction (SPE) of filtered water, pressurized liquid extraction [also called accelerated solvent extraction (ASE)] of SPM, and passive equilibrium sampling (PES) with equilibrium partitioning modeling.

## Materials and Methods

### Sampling

The same sampling sites were chosen as in our previous dry weather study^16^ along the Ammer River, which is a tributary of the Neckar River in Southwest Germany. Site A (48°33′56″N, 8°53′49″E) was located upstream of a wastewater treatment plant (WWTP),^16^ while site B (48°31′34.7″N, 8°57′50.9″E) was 7.8 km downstream of this WWTP. The sampling campaign was conducted on a wet day with a precipitation amount of 7.8 mm over 3 h on 3 February 2021. The nearby regions along the river are mainly agricultural and urban areas.

Surface river water samples were collected in glass bottles at both sites. After being transferred to the laboratory, the SPM was separated from the river water using metal filters (6 μm, 587 cm^2^). While 2000 L of water had to be sampled during dry weather to obtain a sufficient mass of SPM, only 40 L of river water were filtered to obtain a sufficient mass of SPM at site A and 38 L at site B during this sampling campaign. The entire filter cake of SPM was collected, but only 1.7 L of water were processed with SPE. Detailed information on sampling sites and sampling processes can be found in the previous study.

### Extraction of Water Samples

A SPE device with 500 mg HR-X cartridges (Chromabond, Macherey-Nagel, Düren, Germany) was employed to extract and enrich chemicals from 1.7 L of filtered water samples. The cartridges were eluted with ethyl acetate and methanol, and the extracts were reduced to dryness and finally reconstituted in 1.7 mL of methanol with a final enrichment factor of 1000 L_w_/L_methanol_.

### Extraction of SPM Samples

The SPM collected on the filter was freeze-dried and extracted with acetone and ethyl acetate (50:50, v/v) with a pressurized liquid extraction device (DIONEX ASE 350, Thermo Fisher Scientific). The extracts were concentrated and prepared in dichloromethane for further cleanup. A preconditioned silica gel column (Chromabond Flash RS 4 SiOH, Macherey-Nagel, Düren, Germany) was used to clean up the extracts. The eluate was finally prepared in two aliquots; i.e., one aliquot was redissolved in ethyl acetate for GC analysis, and the other in methanol for LC analysis and bioassay dosing. Process and matrix controls were involved during the whole experiment. The analytical methods for water and particle analysis were detailed in our previous study.^16^

The organic carbon (OC)-bound chemicals in SPM were extracted using PES with polydimethylsiloxane (PDMS) according to the method established in Niu et al.^16^ Around 74 mg of precleaned PDMS sheets (0.6 mm thickness) were added into the SPM slurry (total of 10 mL) with 0.1% sodium azide (NaN_3_). The mass ratios of PDMS (*m*_PDMS_) to SPM (*m*_SPM_) in these experiments ranged from 0.211 to 0.213 g_PDMS_/g_SPM,dw_. After shaking at 200 rpm for 50 days, the PDMS sheets were removed, wiped, dried, and extracted twice with ethyl acetate. The extracts were further concentrated and redissolved in ethyl acetate before instrumental analysis. Blanks without an SPM slurry were prepared and processed alongside the field samples.

### Organic Carbon Quantification

The dissolved organic carbon [DOC] and the OC content in SPM [OC, SPM] were determined, as previously described, using a total organic carbon (TOC) analyzer.^16^ The water sample was further filtered with a 0.45 μm cellulose acetate filter before the DOC measurement. In addition, the mass concentration of SPM in the river water [SPM] at each site was calculated based on the volume of filtered water and mass of collected SPM (Table 1).

**Table 1:** Concentrations of Dissolved Organic Carbon [DOC] and Suspended Particulate Matter [SPM] in Water, Organic Carbon Content of SPM [OC, SPM], and OC in Total Water Column ([OC] = [SPM] × [OC, SPM]) in River Samples Collected during Wet and Dry Weather

		wet weather		dry weather^a^	
concentration	unit	site A	site B	site A	site B
[DOC]	(mg_OC_/L)	3.72	4.28	1.63	2.33
[SPM]	(mg_SPM,dw_^b^/L)	421	94.4	1.05	0.95
[OC, SPM]	(mg_OC_/g_SPM,dw_)	50.6	59.9	105	112
[OC]	(mg_OC_/L)	21.5	5.66	0.12	0.11

a Data from Niu et al.^16^;

b dw: dry weight.

### Target Chemical Analysis

Liquid chromatography–high-resolution mass spectrometry (LC–HRMS, Thermo Ultimate 3000 coupled to a Thermo QExactive Plus) and gas chromatography–HRMS (GC–HRMS, Thermo Trace 1310 coupled to a Thermo QExactive GC) were used for the quantification of target chemicals. Instrumental conditions are detailed in our previous studies.^16,18^ Only the 415 target compounds listed in Table S1 were quantified to allow for a direct comparison between dry and wet weather. These compounds covered 15 categories including pharmaceutical and personal care products (PPCPs), pesticides, industrial chemicals, plastic additives, perfluorinated compounds (PFCs), food ingredients, human metabolites, natural compounds, organochlorine pesticides (OCPs), polycyclic aromatic hydrocarbons (PAHs), polybrominated diphenyl ethers (PBDEs), polychlorinated biphenyls (PCBs), pyrethroids, chlorobenzenes, and other halogenated compounds (Table S1). These chemicals are further categorized into two groups: one is named as neutral and hydrophobic chemicals that consist of neutral chemicals with log *K*_ow_ ≥ 3; the other is named as charged or hydrophilic chemicals that consist of charged chemicals or neutral chemicals with log *K*_ow_ < 3 (Table S1).

### Data Evaluation of Partitioning Processes

**Figure 1:**
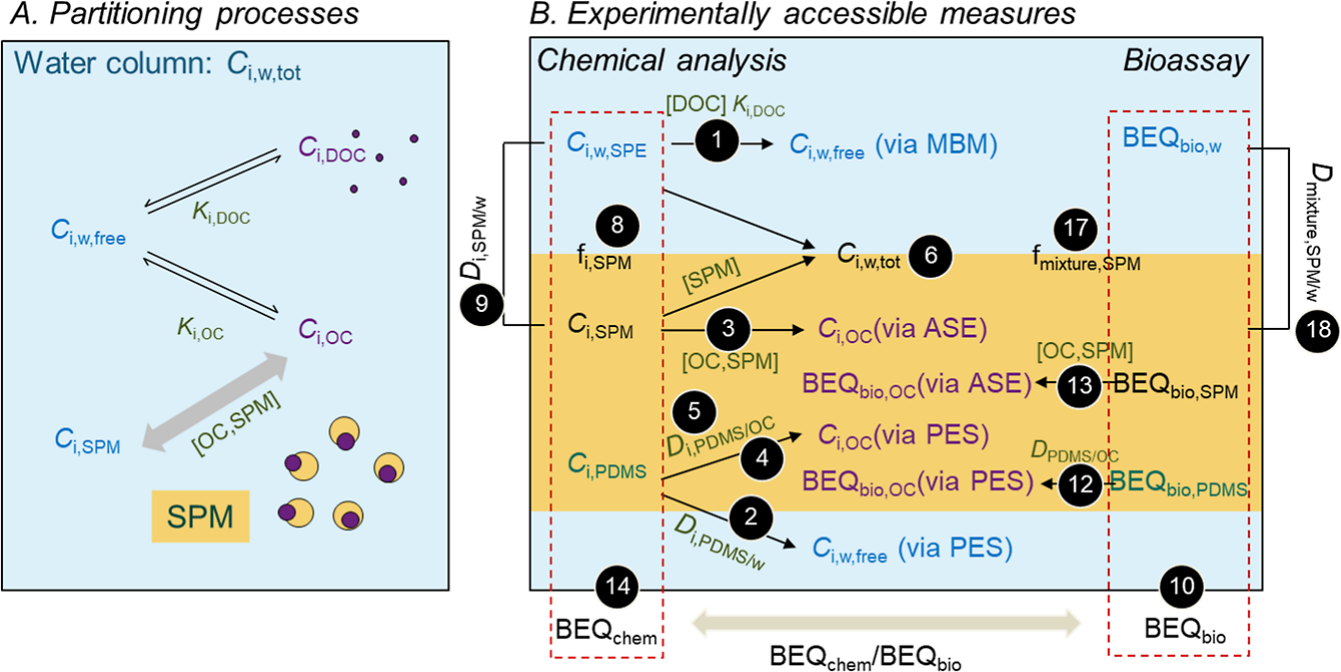
(A) Partitioning processes of chemical i between water (w) and suspended particulate matter (SPM) in a river system and (B) experimentally accessible measures to describe the processes. *C*: concentration; [DOC]: concentration of dissolved organic carbon in the water phase; [OC, SPM]: organic carbon (OC) content of SPM; SPE: solid phase extraction; MBM: mass-balance model; ASE: accelerated solvent extraction; *K*: partitioning constant; *D*: distribution ratio; PES: passive equilibrium sampling; PDMS: polydimethylsiloxane; OC: organic carbon; BEQ_bio_: bioanalytical equivalent concentration measured with the bioassays in the extracts. All abbreviations are detailed in Table S2, and equations are derived in the Materials and Methods section with black dots giving the equation number. The figure was adapted from ref (16) Copyright 2021 American Chemical Society.

All parameters measured and used for calculations are given in Table S2 and summarized in Figure 1. Chemicals in the filtered water phase extracted by SPE (*C*_i,w,SPE_, ng/L_w_) consist of freely dissolved and DOC-bound fractions. The freely dissolved concentration of chemical i in the water (*C*_i,w,free_) can be derived with the mass balance model (MBM) given in eq 1.^16^
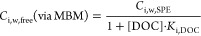
1where *K*_i,DOC_ is the partition constant of chemical i between DOC and water. Because *K*_i,DOC_ is not available, *K*_i,DOC_ was assumed to be equal to *K*_i,OC_, which implicitly assumes the same sorptive capacity of DOC as that of OC. *K*_i,OC_ were previously collected from literature with experimental preferred over predicted data (Table S1).^18^ The *K*_i,OC_ of partially charged chemicals were not corrected for speciation.^18^

*C*_i,w,free_ can also be accessed through the PES from the concentration measured in PDMS (*C*_i,PDMS_, ng/kg_PDMS_) (eq 2).
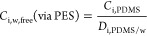
2

*D*_i,PDMS/w_ is the distribution ratio of chemical i between PDMS and water at pH 7 (Table S1) and refers to either previously measured experimental *D*_i,PDMS/w_ at pH 7^19^ or the partition constant *K*_i,PDMS/w_ of the neutral species of chemical i between PDMS and water normalized to the fraction of neutral species at pH 7 for partially ionized chemicals. It is a consensus that ionizable organic chemicals could not or only partly partition into PDMS, depending on the proportion of neutral and charged species.^19^

*C*_i,OC_ is the concentration of chemical i bound to OC (ng/kg_OC_), which can be accessed either from the measured concentration in SPM after ASE, i.e., *C*_i,SPM_ (ng/g_SPM,dw_), considering its OC content [OC,SPM] (g_oc_/g_SPM,dw_) (eq 3), or indirectly via PES (eq 4).
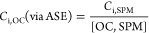
3
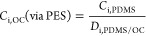
4

*D*_i,PDMS/OC_ is the apparent distribution ratio of chemical i between PDMS and OC, which is the ratio of *D*_i,PDMS/w_ and *D*_i,OC_ (eq 5 and Table S1). As the ionization-corrected *D*_i,OC_ was not available, we used *K*_i,OC_ as a proxy, assuming equal binding affinity for neutral and charged chemicals to OC.
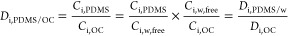
5

The total concentration of chemical i in the water column (*C*_i,w,tot_, ng/L_w_) is the sum of the freely dissolved concentration plus the fraction bound to DOC, which together can be measured as *C*_i,w,SPE_, plus the fraction bound to SPM.
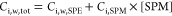
6

The bioavailable fraction of individual compounds, i.e., the freely dissolved fraction (*f*_i,free_), can be computed via *C*_i,w,free_ and the measured dissolved concentration *C*_i,w,SPE_.
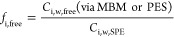
7

To evaluate the contribution of SPM-bound chemicals to the total water column, the fraction in SPM (*f*_i,SPM_, eq 8) can be calculated from the mass concentration of SPM ([SPM]) and the experimental distribution ratio between SPM and water *D*_i,SPM/w_ (L_w_/kg_SPM,dw_, eq 9), where *m*_SPM_ refers to the mass of SPM and *V*_W_ to the volume of water.
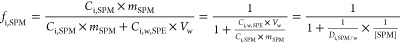
8
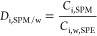
9

### Bioanalysis

Three in vitro bioassays targeting the activation of AhR (AhR CALUX), binding to PPARγ (PPARγ GeneBLAzer), and oxidative stress response (AREc32) were employed in this study for toxicity testing of all types of sample extracts. The cell lines and the testing procedures are detailed in Neale et al.^20^ and König et al.^21^ In addition to specific effects, the cell viability (i.e., cytotoxicity) was simultaneously recorded to exclude false positive responses. The quality control chemicals were 2,3,7,8-TCDD for AhR CALUX, rosiglitazone for PPARγ GeneBLAzer, and *t-*butylhydroquinone for AREc32, with their effect data given in Table S3.

### Data Evaluation for Bioassays and Mixture Effect Modeling

Iceberg modeling was applied to identify the effect drivers in the chemical mixture of sample extracts regarding the targeted modes of action. The mixture effect estimated based on bioassays was expressed as bioanalytical equivalent concentration BEQ_bio_ (ng_ref_/L_w_ for water, μg_ref_/g_SPM,dw_ for SPM, and μg_ref_/g_PDMS_ for PDMS) (eq 10), and the cytotoxicity (toxic unit TU, eq 11) was expressed as TU_bio_ (L_bioassay_/L_w_ or L_bioassay_/kg_SPM,dw_ or L_bioassay_/kg_PDMS,dw_).
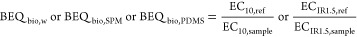
10
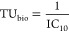
11

EC_10,ref_ (kg_ref_/L_bioassay_) and EC_10,sample_ (kg/L_bioassay_ or L_w_/L_bioassay_) are the concentrations of reference compound and sample, respectively, causing 10% of the maximum effect in the AhR and PPARγ bioassays; EC_IR1.5,ref_ (kg_ref_/L_bioassay_) and EC_IR1.5,sample_ (kg/L_bioassay_ or L_w_/L_bioassay_) are the concentrations of reference compound and sample, respectively, causing an induction ratio of 1.5 in the AREc32 bioassay; and IC_10_ (kg/L_bioassay_ or L_w_/L_bioassay_) is the concentration of sample, causing 10% of cytotoxicity. The concentration of the chemical mixture in the extract is given as relative extraction factor (REF, L_w_/L_bioassay_, kg_SPM_/L_bioassay_ or kg_OC_/L_bioassay_). Benzo[*a*]pyrene was the reference compound for the calculation of BEQ in AhR CALUX, rosiglitazone in PPARγ GeneBLAzer, and dichlorvos in AREc32, as used previously (Table S3). More details on data evaluation can be found in Escher et al.^22^ and Neale et al.^7^

As the apparent partition constant of the chemical mixture between PDMS and OC (*D*_PDMS/OC_) is theoretically independent of the type of chemicals, BEQ_bio,PDMS_ (μg_ref_/g_PDMS_) measured in the PDMS extract can be converted to the BEQ of OC-bound chemical mixture BEQ_bio,OC_ (μg_ref_/g_OC_) by eq 12. A *D*_PDMS/OC_ of 1 for the chemical mixture was estimated in previous work.^23^ The empirical *D*_i,PDMS/OC_ values in Table S1 also come close to 1, with a mean of 1.16 (95% CI 0.93–1.93). As we do not know if the detected chemicals are representative of the mixture composition, and to be consistent with previous work,^23^ we adopted a *D*_PDMS/OC_ of 1 in this study.
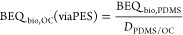
12

BEQ_bio,OC_ can also be derived from the ASE method using the BEQ of the SPM-bound chemical mixture (BEQ_bio,SPM_) and the [OC,SPM].
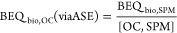
13

The mixture effect of the quantified chemicals (BEQ_chem_, mol/kg_SPM_, mol/kg_OC_, or mol/L_w_, eq 14) can be computed from the chemical concentrations *C*_i_ (mol/kg_SPM_, mol/kg_OC_, or mol/L_w_), weighted by the relative effect potency REP_i_ of chemical i (eq 15). The mixture cytotoxicity TU_chem_ (L_bioassay_/kg or L_bioassay_/L_w_) of the quantified chemicals can be estimated with eq 16.
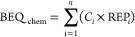
14
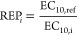
15
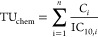
16

EC_10,i_ (kg/L_bioassay_) is the concentration of chemical *i* causing 10% of the maximum effect, and IC_10,i_ (kg/L_bioassay_) is the concentration of chemical i causing 10% of the maximum inhibitory concentration. Among the analyzed chemicals, the REP values are available for 91, 34, and 73 active compounds in AhR CALUX, PPARγ GeneBLAzer, and AREc32 bioassays, respectively, while the corresponding cytotoxicity data are available for 319, 302, and 312 compounds, respectively (Table S3).

The fraction of effect and cytotoxicity explained by the detected chemicals could be estimated by using BEQ_chem_/BEQ_bio_ and TU_chem_/TU_bio_. The contribution of individual chemical i to the total effect and cytotoxicity was evaluated by BEQ_chem,i_/BEQ_chem_ and TU_chem,i_/TU_chem_, respectively.

The contribution of the SPM-bound chemical mixture to the total water column corresponding to specific effects *f*_mixture,SPM_ can be calculated using eq 17 based on the distribution of mixture effect between SPM and water, *D*_mixture,SPM/w_ (L_w_/kg_SPM,dw_, eq 18).
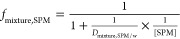
17
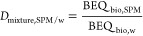
18

## Results and Discussion

### Chemicals in the Water

**Figure 2:**
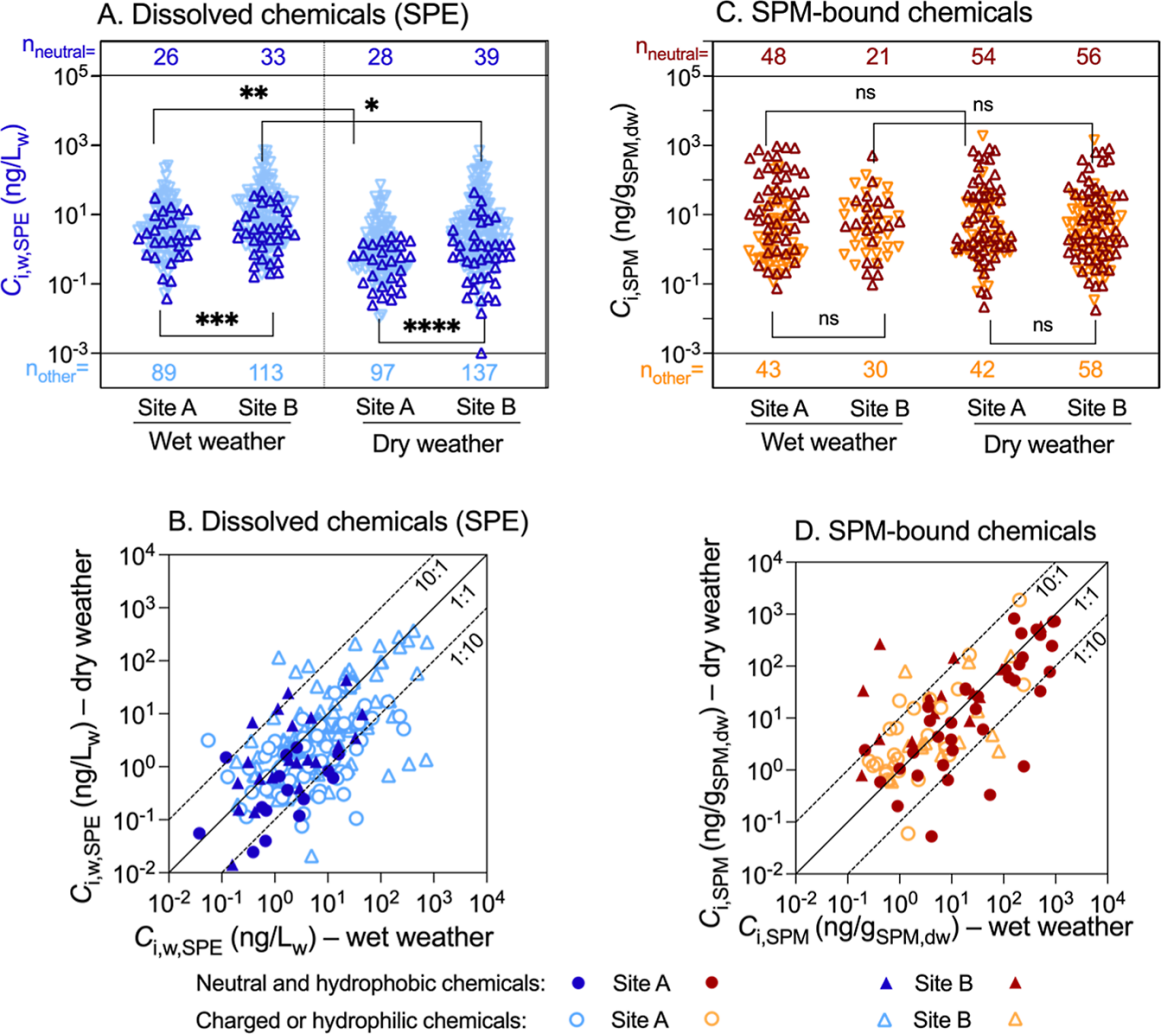
(A) Concentrations of dissolved chemicals (*C*_i,w,SPE_) in the river under wet and dry weather conditions quantified after solid phase extraction (SPE). (B) Comparison of *C*_i,w,SPE_ between wet and dry weather. (C) Concentrations of chemicals in suspended particulate matter (*C*_i,SPM_) under wet and dry weather conditions quantified with accelerated solvent extraction (ASE). (D) Comparison of *C*_i,SPM_ between wet and dry weather. (SPM: suspended particulate matter; n_neutral_ refers to the number of neutral and hydrophobic chemicals, and n_other_ refers to the number of charged or hydrophilic chemicals.) Data in Table S4.

In the water samples, 154 and 181 compounds were detected at least once during wet and dry weather, respectively (Table S4), and measured concentrations in water extracts after SPE were log-normally distributed in all samples (Figure 2A). The concentrations *C*_i,w,SPE_ of target chemicals were up to 271 ng/L_w_ at site A and 743 ng/L_w_ at site B during rainy weather, while they were up to 74.4 ng/L_w_ at site A and 723 ng/L_w_ at site B during dry weather (Table S4). The rain events significantly elevated the total chemical concentrations in the water (Figure 2A, paired *t*-test *p* = 0.0053 for site A and *p* = 0.0298 for site B (all chemicals)) with the sum of molar concentrations increasing from 1.61 to 6.77 nmol/L_w_ at site A and from 17.3 to 25.8 nmol/L_w_ at site B. There was a significant difference in the concentrations between the two sites (Figure S1A,B, paired *t*-test *p* = 0.0001 (wet) and *p* = 0.0002 (dry)). Charged or hydrophilic chemicals had overall higher concentrations than neutral and hydrophobic chemicals in the water phase (Figures 2A and S1). Aqueous concentrations measured by SPE rarely varied by more than a factor of 10 between wet and dry weather (Figure 2B) with a tendency of all chemicals being higher in wet weather at site A (*p* = 0.053) and site B (*p* = 0.0298) (Figure 2A).

Approximately 60% of the chemicals detected in the water at site A showed higher concentrations in wet weather than in dry weather, with most of them being charged or hydrophilic compounds (Figure 2B). At site B, the concentrations of chemicals detected between the two cases were more diverse, with higher numbers of charged or hydrophilic chemicals beyond a 10-fold variation (Figure 2B). More details are given in Text S1 and Figure S2.

### Chemicals Bound to SPM

Compared to those in the water phase, more compounds were detected in SPM samples during dry weather than wet weather (numbers of 96 vs 91 at site A and 114 vs 51 at site B, Figure 2C). The concentrations of target chemicals in bulk SPM detected during the rain events were up to 960 ng/g_SPM,dw_ at site A and 520 ng/g_SPM,dw_ at site B (Figure 2C), while those detected in dry weather were up to 1879 ng/g_SPM,dw_ at site A and 1411 ng/g_SPM,dw_ at site B. The paired *t* tests between sites and weather conditions showed that pairing was highly significant (*p* < 0.0001 for wet vs dry at sites A and B and site A vs B in wet and dry weather; Figure 2C). The sum molar concentrations in bulk SPM were similar at site A (34.2 vs 36.9 nmol/g_SPM,dw_, wet vs dry weather), whereas those at site B were 6-fold lower during rainy weather (5.25 vs 31.3 nmol/g_SPM,dw_). Sampling was performed at different time points, with lower OC content of SPM under wet conditions, resulting in a reduced affinity of pollutants to SPM at site B. However, the distributions did not show any significant differences between wet and dry weather at either site (paired *t*-test, *p* = 0.87 wet vs dry for site A and *p* = 0.09 wet vs dry for site B; Figure 2D).

In terms of site-specific loadings, the total chemical concentrations in SPM collected in dry weather were similar between sites, while in wet weather, concentrations at site A were six times higher than those at site B (Figure S1C,D). More details are provided in Text S2 and Figure S3.

When looking at individual compounds, we found that the variation in total chemical loading between sites during rain events was mainly caused by PAHs, with levels of 27.7 nmol/g_SPM,dw_ at site A and 0.15 nmol/g_SPM,dw_ at site B (Figure S3A). Urban runoff often contains large amounts of PAHs that come from traffic activities such as tire wear, asphalt abrasions, and incomplete combustion of vehicle exhausts.^9,24^ This could account for the chemical variations between sites as site A is located near a highway. However, overflow from WWTPs appears to be an important source of organic chemicals in the case of site B, similar to what has been reported during storm events.^9,13^ On the other hand, rainfall will also dilute the river and might promote the transfer of chemicals from the particle to the water phase, leading to a desorption of SPM-bound contaminants into the water. An enhanced migration to the dissolved phase caused by rainfall was reported for organochlorine pesticides with low *K*_ow_ in the Wang Lake Wetland, China.^25^

### Comparison of Freely Dissolved and OC-Bound Concentrations Measured with Different Methods

**Figure 3:**
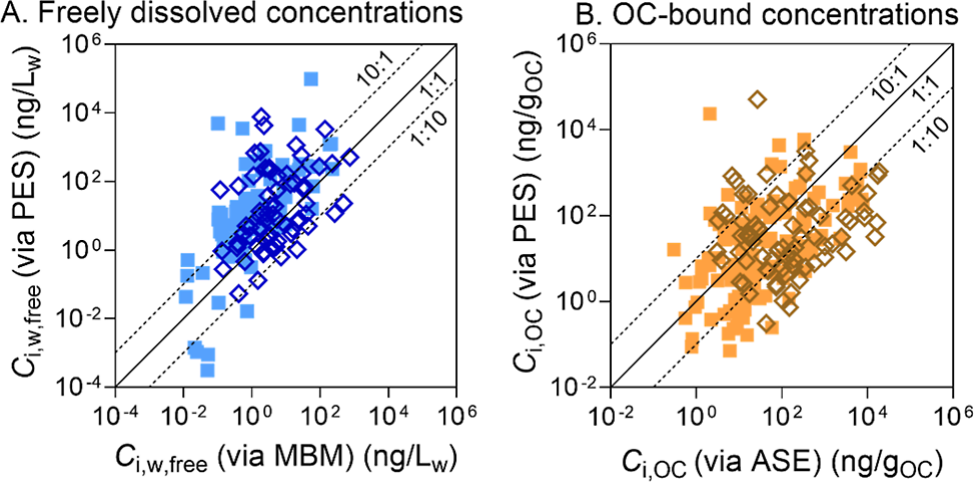
(A) Comparison of freely dissolved concentrations (ng/L_w_) derived with the mass balance model (MBM) from measured concentrations after solid phase extraction [*C*_i,w,free_ (via MBM)] and passive equilibrium sampling [*C*_i,w,free_ (via PES)] and (B) comparison of organic carbon (OC)-bound chemical concentrations (ng/g_OC_) measured by accelerated solvent extraction [*C*_i,OC_ (via ASE)] and PES [*C*_i,OC_ (via PES)]. The empty symbols represent neutral and hydrophobic chemicals, and filled symbols represent charged or hydrophilic chemicals.

The freely dissolved concentration *C*_i,w,free_ (Table S5) can be calculated using the MBM (eq 1) or derived by PES in the SPM slurry (eq 2). The agreement between the two approaches to obtain *C*_i,w,free_ is not very good (Figure 3A), with only 56% of the data within the factor of 10. If chemicals that are charged or hydrophilic and therefore potentially problematic for PES are removed, the correlation between MBM and PES improves, with 65% of the data within the factor of 10 (Figure S4A).

This discrepancy between the two methods for deriving *C*_i,w,free_ is presumably due to the uncertainty in *K*_i,DOC_, which is substituted by *K*_i,OC_ from the literature, and not corrected for speciation in the case of ionizable chemicals. This would lead to an overestimation of the fraction bound to DOC and hence an underestimation of *C*_i,w,free_ by the MBM. *K*_i,OC_ is not a pure compound property but is influenced by the composition and quality of the organic matter, which may easily lead to variation of *K*_i,OC_ by more than an order of magnitude.^26^

In contrast, the *C*_i,w,free_ from PES appears to be more reliable, especially given that we experimentally determined the *D*_i,PDMS/w_ of some ionizable chemicals in this chemical test set in a previous study^19^ using the very same PDMS batch that was used here for PES. The downside is that many fewer chemicals were detected in PDMS (Table S4).

The PES- and ASE-based approach yielded up to 2 orders of magnitude deviation of *C*_i,OC_ from the 1:1 line (Figure 3B and Table S5) and thus showed even more variability between the two methods than *C*_i,w,free_ (Figure 3A). The OC content determined by the TOC analyzer was the amount of total OC in the SPM, which is composed of amorphous OC and black carbon (BC). However, the OC-bound chemicals accessed by PES were mainly those bound to amorphous OC since BC typically decreases the bioavailability of chemicals.^27^ The overestimation of OC resulted in an underestimation of *C*_i,OC_ by the ASE method. In addition, due to the use of *K*_i,OC_ of the neutral species as a substitute for *D*_i,OC_ for ionizable chemicals, the *C*_i,OC_ derived from PES might be overestimated since the *D*_i,PDMS/w_ of some ionizable chemicals used for calculation were the experimentally determined data. The uncertainties in both *K*_i,OC_ and OC content led to a larger divergence of *C*_i,OC_ between these two methods.

### Role of Water DOC and SPM OC in Chemical Concentrations between Wet and Dry Weather

Although the [DOC] in the water was twice as high under wet conditions compared to dry weather, its influence on *C*_i,w,free_ between MBM and PES methods could be excluded because SPE concentrations had been converted to freely dissolved concentrations using eq 1. Guo et al.^28^ found that the DOC content in water significantly influences the partitioning of the herbicide atrazine and its degradation products between the water and SPM phases, with the apparent partition coefficient decreasing along with increasing [DOC], which is consistent with our findings.

The freely dissolved fraction *f*_i,free_ (eq 7) calculated from the SPE extracts was close to 1 for most of the charged and hydrophilic chemicals, while it can be much smaller for very hydrophobic neutral chemicals. Most of the chemicals with a computed small *f*_i,free_ were below their detection limits (MDL, Table S4). The *f*_i,free_ calculated from the PES did not lead to any meaningful results because the *C*_i,w,free_ values (via PES) were often higher than *C*_i,w,SPE_. The *C*_i,w,SPE_ values were not corrected for recovery and could therefore underestimate the true aqueous concentrations. The high signal background and strong matrix effects caused by the complexity of the samples might be responsible for the low recoveries of charged chemicals as previously reported.^29,30^

### Toxicological Profiles of Chemical Mixtures in the Water and SPM

**Figure 4:**
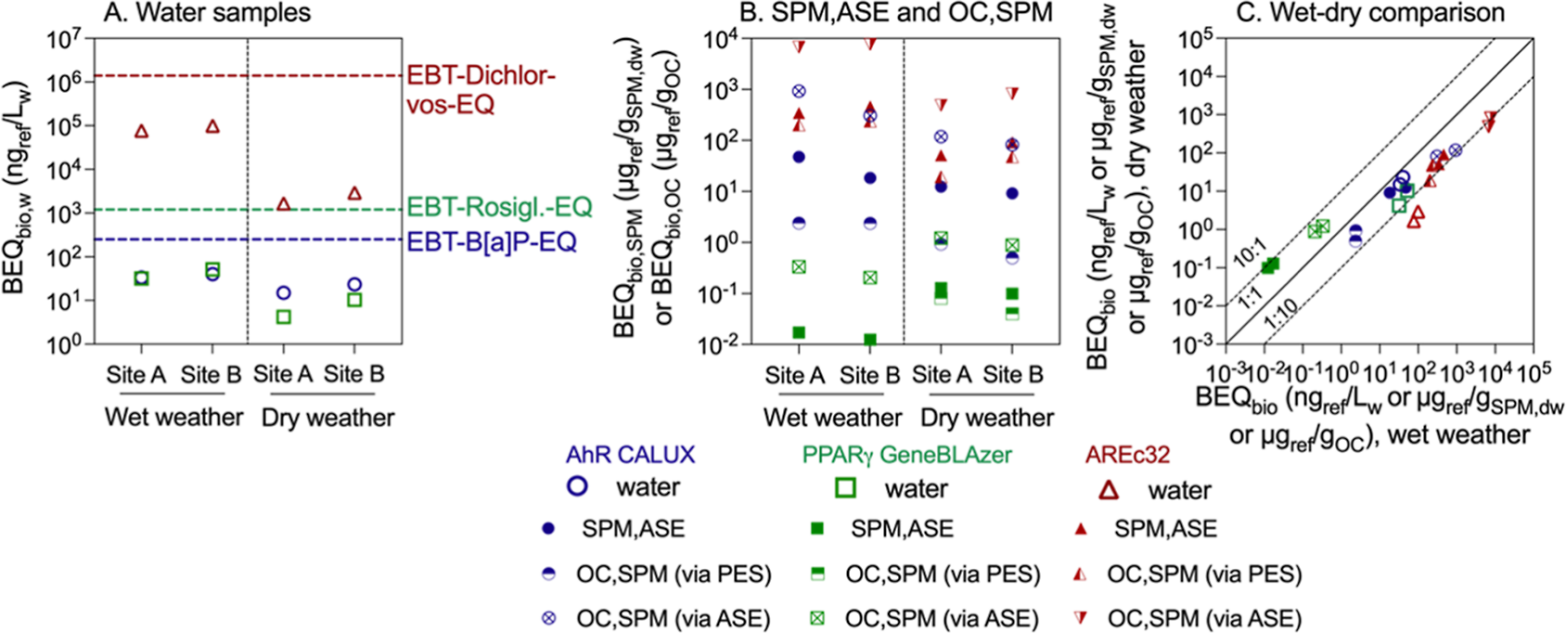
Bioanalytical equivalent concentrations (BEQ_bio_) of chemical mixtures in the (A) water (ng_ref_/L_w_) measured with solid phase extraction (SPE) and (B) suspended particulate matter (ng_ref_/g_SPM,dw_) and the organic carbon (OC) fraction (ng_ref_/g_OC_) that were derived from passive equilibrium sampling (PES) and accelerated solvent extraction (ASE). (C) Comparison of BEQ_bio_ between wet and dry weather. Reference compounds for different bioassays are benzo[*a*]pyrene (B[*a*]P-EQ_bio_) for AhR CALUX, rosiglitazone-EQ_bio_ for PPARγ GeneBLAzer, and dichlorvos-EQ_bio_ for AREc32. Lines in (A) refer to effect-based trigger values (EBT), namely, EBT-B[*a*]P-EQ = 250 ng/L_w_, EBT-rosiglitazone-EQ = 1.2 μg/L_w_, and EBT-dichlorvos-EQ = 1.4 mg/L_w_.^31^ Data in Table S6.

The in vitro bioassays are powerful tools to characterize the risk potential of mixture extracts.^7,21^ The IC_10_ and EC_10_ or EC_IR1.5_ of chemical mixtures in the water, SPM, and PDMS extracts tested in AhR CALUX, PPARγ GeneBLAzer, and AREc32 are summarized in Table S6, and the calculated BEQ_bio_ are visualized in Figure 4. The activation of AhR and oxidative stress response were the most responsive end points for all types of sample extracts, while the binding to PPARγ was not activated by some SPM-related samples. Discussion related to cytotoxicity expressed as TU_bio_ is provided in Text S3 and Figure S5.

The B[*a*]P-EQ_bio_ of the water samples were 33.7 and 40.3 ng/L_w_, rosiglitazone-EQ_bio_ were 31.9 and 51.8 ng/L_w_, and dichlorvos-EQ_bio_ were 76.3 and 99.5 μg/L_w_ at sites A and B, respectively, during wet weather. In the case of dry weather, B[*a*]P-EQ_bio_ were 15.1 and 23.4 ng/L_w_, rosiglitazone-EQ_bio_ were 4.20 and 10.3 ng/L_w_, and dichlorvos-EQ_bio_ were 1.66 and 2.93 ng/L_w_ at sites A and B, respectively. The BEQ_bio_ values were slightly higher at site B than site A (Figure 4A) in analogy to chemical analysis, but larger differences were observed between dry and wet weather. The higher chemical load and effects found at site B are reasonable since it is located downstream of a WWTP, a primary input source impacting the pollution and toxicity of the river.^4,9^ The effect levels of water samples collected during wet weather in the present study were within the ranges observed in small streams of Germany, where the B[*a*]P-EQ_bio_ ranged from 2.4 to 500 ng/L_w_, rosiglitazone-EQ_bio_ ranged from 2.4 to 628 ng/L_w_, and dichlorvos-EQ_bio_ ranged from 18.4 to 2985 μg/L_w_ during rain events.^7^ The BEQ_bio_ values of water in all bioassays were below the effect-based trigger values (EBT-B[*a*]P-EQ = 250 ng/L_w_ for AhR CALUX, EBT-rosiglitazone-EQ = 1.2 μg/L_w_ for PPARγ GeneBLAzer, and EBT-dichlorvos-EQ = 1.4 mg/L_w_ for AREc32).^31^

The differences in BEQ_bio_ between wet and dry weather were more pronounced than between sites but depended very much on the bioassay end points (Figure 4A). The B[*a*]P-EQ values of water mixtures collected under the two conditions were very close to each other, whereas the rosiglitazone-EQs and dichlorvos-EQs were both higher during rain events (Figure 4A). Similarly, higher effects in water were found in small streams of Germany during rainfall events, which has been proven to be influenced by the overflow from water retention basins or sewer systems.^7^ Knauer et al.^32^ put forward that only the toxicity of pyrethroid insecticides with log *K*_ow_ > 5 could be decreased due to the presence of SPM in surface water, whereas pesticides with log *K*_ow_ < 3 were hardly impacted. On the other side, it has been reported that the increasing occurrence of SPM by sediment perturbation and other inputs caused by rainfall may enhance the sorption of more hydrophobic organic chemicals and thus decrease their dissolved concentrations and associated effects in the water.^33,34^

ASE appeared to yield much higher BEQ_bio,OC_ than PES for the corresponding samples in AhR CALUX, while they were similar for PPARγ-GeneBLAzer and AREc32 (Figure 4B and Table S6). PES only accesses the readily desorbing (bioaccessible) fraction of contaminants,^35^ while ASE also extracts strongly bound fractions.^36^

The B[*a*]P-EQ_bio_ of extracts in bulk SPM increased from 9.14–12.4 μg/g_SPM_ to 18.4–47.4 μg/g_SPM_ by rain events and the dichlorvos-EQ_bio_ from 50.9–91.2 μg/g_SPM_ to 343–459 μg/g_SPM_ (Figure 4B and Table S6). However, the rosiglitazone-EQ_bio_ were decreased from 0.10–0.13 to 0.01–0.02 μg/g_SPM_. Similar changes were also found for the effects of OC-bound mixtures tested by the three bioassays. Generally, the BEQ_bio_ of SPM in PPARγ-GeneBLAzer were higher during dry weather than during wet weather, but for PPARγ-GeneBLAzer regarding water as well as AhR CALUX and AREc32 regarding both SPM and water, the BEQ_bio_ were lower during dry weather (Figure 4C).

### How Do Chemicals and Mixture Effects Distribute between SPM and Water?

**Figure 5:**
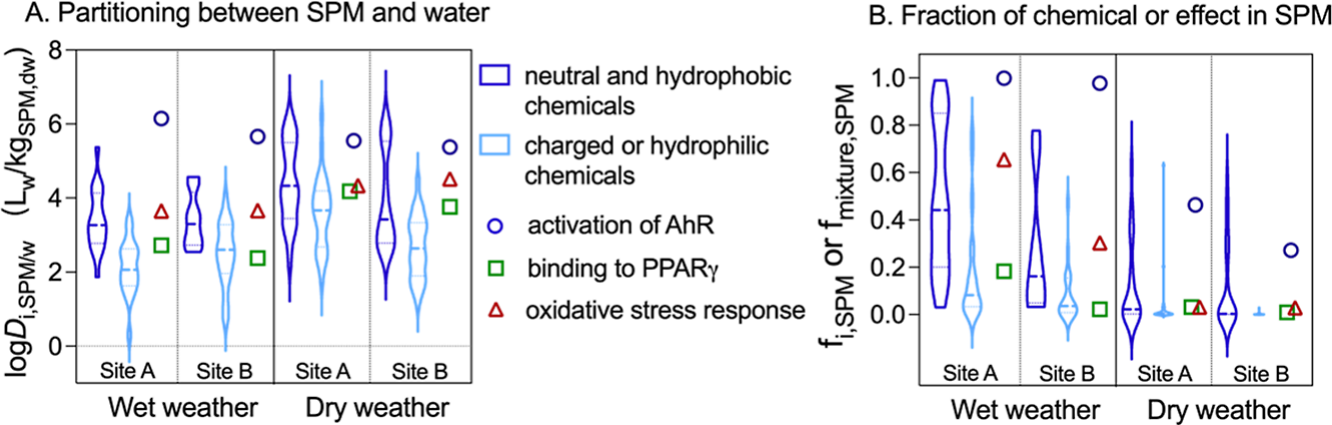
(A) Distribution ratios of individual contaminants (*D*_i,SPM/w_) and chemical mixtures quantified with in vitro bioassays (*D*_mixture,SPM/w_) between the suspended particulate matter (SPM) and water during wet and dry weather. (B) Contribution of individual contaminants (*f*_i,SPM_) and mixture effect (*f*_mixture,SPM_) in SPM to the total chemical load and effect of the entire water column. Bioassays: AhR = arylhydrocarbon receptor; PPARγ = peroxisome proliferator activated receptor gamma. The dashed lines in the violin plots represent the 50th percentiles, and the dotted lines represent the 25th and 75th percentiles of the distribution.

The apparent log *D*_i,SPM/w_ (eq 9) of chemicals detected in wet weather were smaller than those detected in dry weather at site A (median of 2.6 vs 3.9), while those at site B were comparable between the two hydrological conditions (median of 2.8 vs 3.0) (Figure 5A), which can be explained by the smaller [OC, SPM] during wet weather.

The *D*_mixture,SPM/w_ (eq 18) values were calculated from the measured BEQ_bio_ values in paired SPM and water samples. The *D*_mixture,SPM/w_ values in the AhR CALUX were at the high end of the distribution of *D*_i,SPM/w_, of the single chemicals. *D*_mixture,SPM/w_ slightly increased during wet weather (log *D*_mixture,SPM/w_ of 6.1 (site A) and 5.7 (site B) vs 5.5 (both sites), dry weather, Figure 5A).

Like the decreased partitioning between SPM and water phases for neutral and hydrophobic chemicals, decreased *D*_mixture,SPM/w_ was observed for oxidative stress response in wet weather (log *D*_mixture,SPM/w_ of 3.7 vs 4.3–4.5). The *D*_mixture,SPM/w_ derived from the BEQ_bio_ in the PPARγ bioassay [log *D*_mixture,SPM/w_ of 2.4–2.7 (wet) vs 3.8–4.2 (dry)] followed the *D*_i,SPM/w_ trend of charged or hydrophilic chemicals, which can be explained by the fact that most chemicals activating PPARγ carry a carboxylic acid group and are anionic. During wet weather, the *D*_mixture,SPM/w_ of AhR CALUX exceeded the highest *D*_i,SPM/water_ of detected chemicals by 1 order of magnitude. This indicates that neutral and hydrophobic chemicals may play a larger role in mixture toxicity than that expected from chemical analysis.

### Impact of SPM on Organic Contaminants in Rivers under Different Hydrological Conditions

The significance of SPM in acting as an effective vector for the transport and risks of contaminants in river systems has been highlighted in multiple cases, particularly during rain events. Glaser et al.^13^ found that over 50% of the total cytotoxicity in the water column (water plus SPM) was attributable to bioactive chemicals bound to particles when the SPM concentration was beyond 0.5 g/L. Müller et al.^12^ demonstrated the importance of SPM-facilitated chemicals in the overall risk of rivers during rain events as the SPM flux had a dominant effect on the entire water column.

For single chemicals, the fractions held in SPM (*f*_i,SPM_, eq 8) in wet weather contributed from 0.001 to 0.99 at site A and from 0.001 to 0.78 at site B to the total chemical concentrations in the water column, with the median *f*_i,SPM_ values of 0.16 at site A and 0.06 at site B (Figure 5B). Even though the chemical concentrations in SPM collected in wet weather were similar to or lower than those in dry weather, the median *f*_i,SPM_ values were 18–63 times higher because of the high [SPM] in rivers either introduced by runoff or resuspended from the sediment. A similar elevated contribution was observed for azithromycin and levofloxacin with an increasing SPM level in the Asano River, Japan.^37^ The *f*_i,SPM_ of charged or hydrophilic chemicals were close to 0 under both weather conditions as expected from their low *D*_i,SPM/w_. However, neutral and hydrophobic chemicals showed an increased contribution to SPM in wet weather (median of 0.44 at site A and 0.16 at site B during wet weather and of 0.02 at site A and 0.003 at site B during dry weather), especially at site A (Figure 5B). It has been reported that more hydrophobic PAHs, which are ubiquitously present in urban runoff, favor partitioning to particulate fractions in stormwater.^38^ In addition, release from resuspended sediment might also contribute since the Ammer River sediment carries a high load of PAHs, as found previously.^13^

When estimating the fractions of SPM-bound mixtures in the total water column (*f*_mixture,SPM_) using bioactivity data (eq 17), a higher contribution was observed in wet weather compared to dry weather across all three tested bioassays (Figure 5B). It is noteworthy that the *f*_mixture,SPM_ values in the AhR bioassay were nearly 1.0 at both sites during rainfall. In addition, the fraction regarding the oxidative stress response at site A reached 0.30–0.65 during wet weather, which was 10–20 times higher than during dry weather. The *f*_mixture,SPM_ values estimated in PPARγ CALUX were the lowest and showed no significant changes, ranging from 0.02 to 0.18 in wet weather and from 0.01 to 0.03 in dry weather (Figure 5B).

### Key Pollutants Driving the Mixture Risks in Rivers during Wet versus Dry Weather

Iceberg modeling was employed to identify key toxicants for the activation of AhR, binding to PPARγ, and the oxidative stress response. Less than 0.01 of TU_bio_ and 0.05 of BEQ_bio_ could be explained by TU_chem_ and BEQ_chem_ in most of the water samples (Table S6 and Figures S6 and S7), similar to other studies targeting these effect endpoints.^4,7^ There were notable exceptions: Chrysene and benz[*a*]anthracene, which are PAHs, were identified as the main toxic components contributing to over 90% of the AhR effect and oxidative stress response in water during both wet and dry weather at site A (Figures S7A and S8 and Table S7). Site A is close to a highway and is impacted by direct road runoff. Even though the concentrations of chrysene and benz[*a*]anthracene were not the highest in the water, their REP values were high (REP = 0.133 for chrysene and 0.07 for benz[*a*]anthracene), making them stand out among all detected pollutants. The contribution of diclofenac, which likely stems from WWTP effluent or combined sewer overflow, to the rosiglitazone-EQs increased from 5.6 to 48% after rainfall at site A (Figures S7B and S8). Chrysene and benz[*a*]anthracene were also the dominant activators of oxidative stress response in SPM, but only a small fraction of the effect was explained in the water samples (Figures S7C and S8).

BEQ_chem_/BEQ_bio_ were even lower for SPM in AhR [0.001–0.06 (wet) vs 0.02–0.03 (dry)] and oxidative stress response [0.001–0.30 (wet) vs 0.31–0.61 (dry)] but comparable for PPARγ [0.001 (wet and dry)] (Figures S7 and S9). The mixture effects in SPM were dominated by fewer toxicants in wet weather than in dry weather (Figure S9). Benzo[*k*]fluoranthene was identified as the main risk driver at site A for the AhR effect and oxidative stress response in SPM after storm events, whereas dibenz[a,h]anthracene was identified at site B due to a combination of high concentration (Table S4) and REP (Table S3). Regarding the PPARγ effect, the contribution of triphenyl phosphate to rosiglitazone-EQ_bio_ increased from 10.8% (dry weather) to 99.7% (wet weather) at site B, which might be associated with the combined sewer overflow.

### Bioassays as Sentinels to Track the Role of SPM

This study shows that the common practice of filtering water before SPE only captures the dissolved concentrations but not the total burden of chemicals. If one is interested in bioavailable fractions, this approach is sufficient, but if one is interested in total contamination, the chemicals bound to SPM should be quantified. As higher [SPM] would clog the SPE cartridge, for practical reasons, water must be filtered. This filter cake should also be extracted, with both extracts combined prior to chemical analysis and bioassays if one is interested in the total chemical burden. For [OC] < 1 mg_OC_/L_w_, only a small fraction of bioactive chemicals would be associated with SPM, and the role of DOC would therefore become relevant. DOC is extracted by SPE and does not clog the column. PES was evaluated to obtain a more precise picture of the bioavailable fraction of chemical mixtures, but the uncertainty of the partition constants between the PDMS and water or SPM is too high to obtain a reliable picture of the true chemical loads of the water column.

The [SPM] values during wet weather were 90–400 times higher than those in dry weather (Table 1). Therefore, it is reasonable to find SPM-bound chemicals as dominant contributors to the chemical loads and effects in the total water column, especially for neutral and hydrophobic chemicals. These compounds have a high affinity to particles^39^ and are the main potent chemicals for the activation of AhR and oxidative stress response (Table S3). A similar high AhR effect attributable to the SPM-carried pollutants was previously found during storm events in the same river.^12^ Considering the sensitivity of AhR activity to the changes of SPM-bound chemicals and their mixtures, we propose that the AhR activity could serve as an indicator for assessing the risks of SPM-related pollution in rivers.

## Supporting Information Available

Additional information on the variables used in this study, detailed discussion on concentrations detected in water and particles, cytotoxicity-related results and resutls of mixture effect modeling (PDF)
Additional information on the physicochemical properties of the detected chemicals, detailed toxicity data of the detected chemicals and the samples, concentrations of chemicals, calculated freely dissolved and particle-bound concentrations, top mixture effect drivers (XLSX)
