# Supplementary material for: Role of Suspended Particulate Matter for the Transport and Risks of Organic Micropollutant Mixtures in Rivers: A Comparison between Baseflow and High Discharge Conditions
Source: Environ Sci Technol. 2025 Feb 11;59(10):4857–67. doi: 10.1021/acs.est.4c13378 (PMC11924229; doi:10.1021/acs.est.4c13378)
Supplement: Supplementary file 1 — es4c13378_si_001.pdf [file es4c13378_si_001.pdf]

Supporting Information

Role of suspended particulate matter for the transport and risks of organic micropollutant mixtures in rivers: A comparison between baseflow and high discharge conditions

Lili Niu[1,2], Andrea A. E. Gärtner[1], Maria König[1], Martin Krauss[3], Stephanie Spahr[4], Beate I. Escher[1,5]*

[1]Department of Cell Toxicology, Helmholtz Centre for Environmental Research - UFZ, 04318 Leipzig, Germany

[2]Key Laboratory of Pollution Exposure and Health Intervention of Zhejiang Province, Interdisciplinary Research Academy (IRA), Zhejiang Shuren University, 310015 Hangzhou, China

[3]Department of Exposure Science, Helmholtz Centre for Environmental Research - UFZ, 04318 Leipzig, Germany

[4]Department of Ecohydrology and Biogeochemistry, Leibniz Institute of Freshwater Ecology and Inland Fisheries (IGB), Müggelseedamm 301, 12587 Berlin, Germany

[5]Department of Geosciences, Eberhard Karls University of Tübingen, Schnarrenbergstr. 94-96, 72076 Tübingen, Germany

Summary:

18 Pages

7 Tables

9 Figures

3 Text

Table of Contents (xlsx file)

Table S1. Names, structures, and related physicochemical properties (octanol-water partition constant $\log K_{ow}$, fraction neutral species $f_{neutral}$, partition constant between organic carbon and water $\log K_{oc}$ and ionization-corrected distribution ratio between polydimethylsiloxane (PDMS) and water $\log D_{PDSM/w}$ or OC, $\log D_{PDSM/OC}$ of target chemicals analyzed both in wet and dry weather.
Table S3. Bioactivity and toxicity data of target compounds in *in vitro* bioassays indicative of the activation of aryl hydrocarbon (AhR), binding to peroxisome proliferator-activated receptor gamma (PPARγ) and oxidative stress response with AREc32.
Table S4. Directly measured concentrations of detected chemicals in water after solid-phase extraction (SPE), suspended particulate matter (SPM) after accelerated solvent extraction (ASE), and in polydimethylsiloxane (PDMS) after passive equilibrium sampling (PES) in wet and dry weather.
Table S5. Freely dissolved aqueous concentration ($C_{i,w,free}$) and organic carbon (OC)-bound concentrations ($C_{i,OC}$) of detected chemicals i calculated from measured concentrations of detected chemicals in water after solid-phase extraction (SPE), suspended particulate matter (SPM) after accelerated solvent extraction (ASE), and in polydimethylsiloxane (PDMS) (Table S4) via a mass-balance model (MBM, eqs. 1 and 3) or via partition constants to PDMS (eqs. 2 and 4).
Table S6. The effect concentration that caused 10% of the maximum inhibitory concentration (IC_{10}) and 10% of the maximum effect (EC_{10}) or an induction ratio of 1.5 ($EC_{IR1.5}$), the bioanalytical equivalent concentrations (BEQ_{bio} and BEQ_{chem}) and toxic unit for cytotoxicity (TU_{bio} and TU_{chem}) of chemical mixtures calculated based on effect and chemical concentrations and the fractions of observed effects explained by detected chemicals in water, suspended particulate matter (SPM), and the organic carbon (OC)-bound fraction in SPM.
Table S7. Top 10 risk drivers in chemical mixtures identified by iceberg modeling and their contributions to BEQ_{chem}.

Table of Contents (this file)

Table S2. All variables used in this study in the alphabetical order. 4
Text S1. Detailed discussion on chemical concentrations in water 9
Fig. S1. Comparisons of chemical concentrations in the water and suspended particulate matter (SPM) between sampling sites during (A and B) wet and (C and D) dry weather. 8
Text S2. Detailed discussion on chemical concentrations in suspended particulate matter......... 11
Fig. S2. (A, C and E) Concentrations and (B, D and F) compositions of different chemical groups in the water phase ($C_{i,w,SPE}$) under wet and dry weather conditions. PPCPs: pharmaceuticals and personal care products; PFCs: perfluorinated compounds; PAHs: polycyclic aromatic hydrocarbons; PCBs: polychlorinated biphenyls; OCPs: organochlorine pesticides; PBDE: polybrominated diphenyl ethers. 10
Fig. S3. (A, C and E) Concentrations and (B, D and F) compositions of different chemical groups in suspended particulate matter (SPM) of water ($C_{i,SPM}$). PPCPs: pharmaceuticals and personal care products; PFCs: perfluorinated compounds; PAHs: polycyclic aromatic hydrocarbons; PCBs:

polychlorinated biphenyls; OCPs: organochlorine pesticides; PBDE: polybrominated diphenyl ethers. 12
Fig. S4 Figure analogous to Figure 2 but for the neutral and hydrophobic chemicals. (A) Comparisons of freely dissolved concentrations (ng/L) derived with the mass balance model from measured concentration after solid phase extraction ($C_{i,free,SPE}$) and passive equilibrium sampling ($C_{i,free,PES}$); and (B) comparisons of organic carbon (OC)-bound chemical concentrations (ng/g_{OC}) measured by accelerated solvent extraction ($C_{i,OC,ASE}$) and PES ($C_{i,OC,PES}$). 13
Text S3. Cytotoxicity of chemical mixtures in the water, suspended particulate matter (SPM), and organic carbon (OC)-bound fraction during wet and dry weather using *in vitro* bioassays. 14
Fig. S5. Toxic units regarding cytotoxicity (TU_{bio}) of chemical mixtures in the (A) water, (B) suspended particulate matter (SPM) and organic carbon (OC) fractions. 15
Fig. S6. Contribution of detected chemicals to the observed cytotoxicity using (A) AhR-CALUX cells, (B) PPARγ-geneBLAzer cells and (C) AREc32 cells. 15
Fig. S7. Contribution of detected chemicals to the observed effects regarding the (A) activation of aryl hydrocarbon receptor activity (AhR), (B) binding to peroxisome proliferator-activated receptor gamma (PPARγ) and (C) oxidative stress response (ratio of bioanalytical equivalent concentration estimated from chemical analysis BEQ_{chem} to those from bioanalysis BEQ_{bio}) 16
Fig. S8. Contribution of individual chemicals to the total bioanalytical equivalent concentrations (BEQ_{chem}) of water samples for (A) activation of aryl hydrocarbon receptor activity (AhR), (B) binding to peroxisome proliferator-activated receptor gamma (PPARγ) and (C) oxidative stress response during wet and dry weather. 17
Fig. S9. Contribution of individual chemicals to the total bioanalytical equivalent concentrations (BEQ_{chem}) of bulk suspended particulate matter (SPM) for (A) activation of aryl hydrocarbon receptor activity (AhR), (B) binding to peroxisome proliferator-activated receptor gamma (PPARγ) and(C) oxidative stress response during wet and dry weather. 18

Table S2. All variables used in this study in the alphabetical order.

Variable	Unit	Annotation	Location
**Concentration**			
C_{i}	mol/g or mol/L_w	Concentration of chemical i in water, suspended particulate matter (SPM) or polydimethylsiloxane (PDMS)	Eq. 14 and 16
$C_{\mathrm{i,OC}}$ (via ASE)	ng/g_{OC}	Concentration of chemical i bound to organic carbon (OC) in SPM directly measured with accelerated solvent extraction (ASE) and normalized to OC content	Eq. 3
$C_{\mathrm{i,OC}}$ (via PES)	ng/g_{OC}	Concentration of chemical i bound to organic carbon (OC) indirectly via passive equilibrium sampling (PES)	Eq. 4
$C_{\mathrm{i,PDMS}}$	ng/g_{PDMS}	Concentration of chemical i partitioned into polydimethylsiloxane (PDMS)	Eq. 2 and 4
$C_{\mathrm{i,SPM}}$	ng/g_{dw}	Concentration of chemical i bound to suspended particulate matter (SPM)	Eq. 3, 6 and 9
$C_{\mathrm{i,w,free}}$ (via MBM)	ng/L_w	Freely dissolved concentration of chemical i in water phase accessed via mass-balance model (MBM) from $C_{\mathrm{i,w,SPE}}$	Eq. 1 and 7
$C_{\mathrm{i,w,free}}$ (via PES)	ng/L_w	Freely dissolved concentration of chemical i in water phase accessed via passive equilibrium sampling (PES) with polydimethylsiloxane (PDMS)	Eq. 2 and 7
$C_{\mathrm{i,w,SPE}}$	ng/L_w	Concentration of chemical i in water phase extracted by solid phase extraction (SPE)	Eq. 1, 6, 7 and 9
$C_{\mathrm{i,w,tot}}$	ng/L_w	Chemical concentration of chemical i in total water column, including dissolved organic carbon (DOC)- and suspended particulate matter (SPM)-bound.	Eq. 6
[DOC]	mg_{OC}/L_w	Concentration of dissolved organic carbon (DOC) in water	Table 1 and eq. 1
[OC]	mg_{OC}/L_w	Organic carbon (OC) content of suspended particulate matter (SPM)	Table 1
[OC, SPM]	$g_{OC}/g_{SPM,dw}$	Organic carbon (OC) content of suspended particulate matter (SPM) in water column	Table 1 and eq. 3 and 13

Variable	Unit	Annotation	Location
[SPM]	$g_{SPM,dw}/L_w$	Concentration suspended particulate matter (SPM) in water column	Table 1, eq. 6, 8 and 17
**Mass and volume**			
m_{PDMS}	g_{PDMS}	Mass of polydimethylsiloxane (PDMS)	Section "Extraction of SPM"
m_{SPM}	g_{dw}	Mass of SPM collected by filtration method	Section "Extraction of SPM"
V_w	L	Volume of water	Eq. 8
dw	g	Dry weight of suspended particulate matter (SPM)	Table 1
**Partition constants**			
$D_{i,PDMS/w}$	L_w/kg_{PDMS}	Ionization-corrected distribution ratio of chemical i between polydimethylsiloxane (PDMS) and water	Eq. 2 and 5
$D_{i,PDMS/OC}$	kg_{OC}/kg_{PDMS}	Ionization-corrected distribution ratio constant between polydimethylsiloxane (PDMS) and organic carbon (OC)	Eq. 4 and 5
$D_{i,SPM/w}$	$L_w/kg_{SPM,dw}$	Distribution ratio of chemical i between suspended particulate matter (SPM) and water	Eq. 8 and 9
$D_{mixture,SPM/w}$	$L_w/kg_{SPM,dw}$	Distribution ratio of chemical mixture between suspended particulate matter (SPM) and water	Eq. 17 and 18
$f_{i,free}$	-	Freely dissolved fraction of chemical i	Eq. 7
$f_{i,neutral}$	-	Neutral fraction of chemical i	Table S2
$f_{i,SPM}$	-	Fraction of the suspended particulate matter (SPM)-bound chemical i in the total water column	Eq. 8
$f_{mixture,SPM}$	-	Fraction of the suspended particulate matter (SPM)-bound chemical mixture in the total water column	Eq. 17
$K_{i,DOC}$	L_w/kg_{DOC}	Partition constant of chemical i between dissolved organic carbon (OC) and water	Eq. 1

Variable	Unit	Annotation	Location
$K_{i,OC}$	L_w/kg_{OC}	Partition constant of chemical i between organic carbon (OC) and water	Eq. 1 and 5, Table S2
$K_{i,ow}$	$L_w/L_{octanol}$	Octanol/water partition coefficient of chemical i	Table S2
$K_{PDMS/OC}$	kg_{OC}/kg_{PDMS}	Partition constant of chemical mixture between polydimethylsiloxane (PDMS) and organic carbon (OC)	Eq. 12
**Bioassays**			
BEQ_{bio}	ng_{ref}/L_w or ng_{ref}/g	Bioanalytical equivalent concentration (BEQ) measured directly in bioassay	Eq. 10
$BEQ_{bio,w}$	ng_{ref}/L_w	Bioanalytical equivalent concentration (BEQ) of chemical mixture in water extracted by solid phase extraction (SPE)	Eq. 10 and 18
$BEQ_{bio,SPM}$	$ng_{ref}/g_{SPM,dw}$	Bioanalytical equivalent concentration (BEQ) of chemical mixture in suspended particulate matter (SPM) extracted with accelerated solvent extraction (ASE)	Eq. 10, 13 and 18
$BEQ_{bio,PDMS}$	ng_{ref}/g_{PDMS}	Bioanalytical equivalent concentration (BEQ) of chemical mixture partitioned into polydimethylsiloxane (PDMS) via passive equilibrium sampling (PES)	Eq. 10 and 12
$BEQ_{bio,OC}$ (via PES)	ng_{ref}/g_{OC}	Bioanalytical equivalent concentration (BEQ) of chemical mixture bound to organic carbon (OC) measured via passive equilibrium sampling (PES)	Eq. 12
$BEQ_{bio,OC}$ (via ASE)	ng_{ref}/g_{OC}	Bioanalytical equivalent concentration (BEQ) of chemical mixture bound to organic carbon (OC) measured with those of suspended particulate matter (SPM) extracted with accelerated solvent extraction (ASE) and converted by the organic carbon (OC) content	Eq. 13
BEQ_{chem}	mol/L_w or mol/g	Bioanalytical equivalent concentration (BEQ) predicted from the detected chemicals and their relative effect potency REP	Eq. 14
$EC_{10,i}$	$kg/L_{bioassay}$	Concentration of chemical i causing 10% of the maximum effect	
$EC_{10,ref}$	$kg_{ref}/L_{bioassay}$	Concentration of reference compound causing 10% of the maximum effect	Eq. 10 and 15

Variable	Unit	Annotation	Location
$EC_{10,sample}$	$kg/L_{bioassay}$ or $L_w/L_{bioassay}$	Concentration of sample mixture causing 10% of the maximum effect	Eq. 10
$EC_{IR1.5,ref}$	$kg/L_{bioassay}$	Concentration of reference compound causing an induction ratio IR of 1.5	Eq. 10
$EC_{IR1.5,sample}$	$kg/L_{bioassay}$ or $L_w/L_{bioassay}$	Concentration of sample mixture causing an induction ratio IR of 1.5	Eq. 10
IC_{10}	$kg/L_{bioassay}$ or $L_w/L_{bioassay}$	Concentration of sample mixture causing 10% of the maximum inhibitory concentration	Eq. 11
$IC_{10,i}$	$kg/L_{bioassay}$	Concentration of chemical i causing 10% of the maximum inhibitory concentration	Eq. 16
REP_i		Relative effect potency	Eq. 14 and 15
TU_{bio}	$L_{bioassay}/g$ or $L_{bioassay}/L_w$	Toxic unit for cytotoxicity measured in bioassay	Eq. 11
TU_{chem}	$L_{bioassay}/g$ or $L_{bioassay}/L_w$	Cytotoxicity toxic unit calculated from detected chemicals	Eq. 16

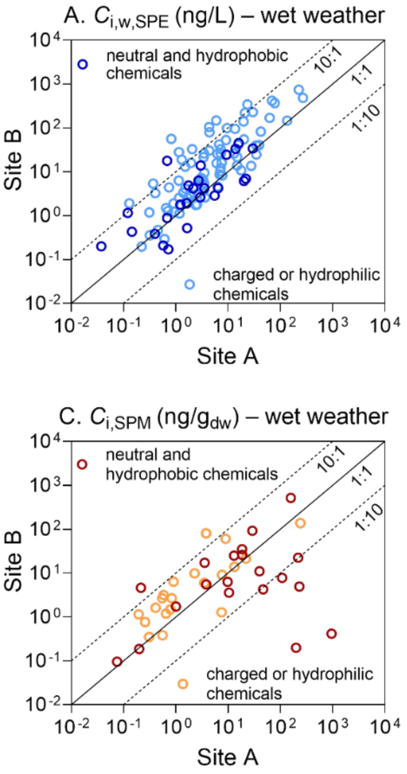

Fig. S1. Comparisons of chemical concentrations in the water and suspended particulate matter (SPM) between sampling sites during (A and B) wet and (C and D) dry weather.

Text S1. Detailed discussion on chemical concentrations in water

As during dry weather, the chemical concentration in water samples collected during wet weather followed the trend of Site B > Site A, but with less variation. Theoretically, the overflow from the WWTP caused by rainfall may exaggerate the difference in chemical burden between Site A and Site B because they are situated on opposite sides of the WWTP. Site B clearly contained more PPCPs, food ingredients and PFCs (Figure S2A). The sum molar concentrations of industrial chemicals, PPCPs and pesticides contributed 85–97% to the total chemical burden in the water phase (Fig. S2B). The contribution of industrial chemicals increased significantly during the rain event, tripling at Site A and doubling at Site B, and became the dominated group at Site A (45%, Fig. S2B). In addition, plastic additives contributed from less than 1% during dry weather to 6.3% during the rain event at Site A, likely because plastic additives are one group of components related to tire wear.[23, 24] Even though pesticides dominated among neutral and hydrophobic chemicals (Fig. S2C), no significant changes were found for their proportions during dry and wet weather (40% at Site A and from 43% to 33% at Site B (Fig. S2D). Charged or hydrophilic chemicals are mainly comprised of PPCPs, industrial chemicals and pesticides, with industrial chemical levels increased by rainfall. Taken together, the above information supports the relevance of road runoff and overflow from WWTP to the river during rain events. Nevertheless, the concentrations did not change very much, but given the higher flow rate, the loads would be expected to differ more between dry and wet weather.

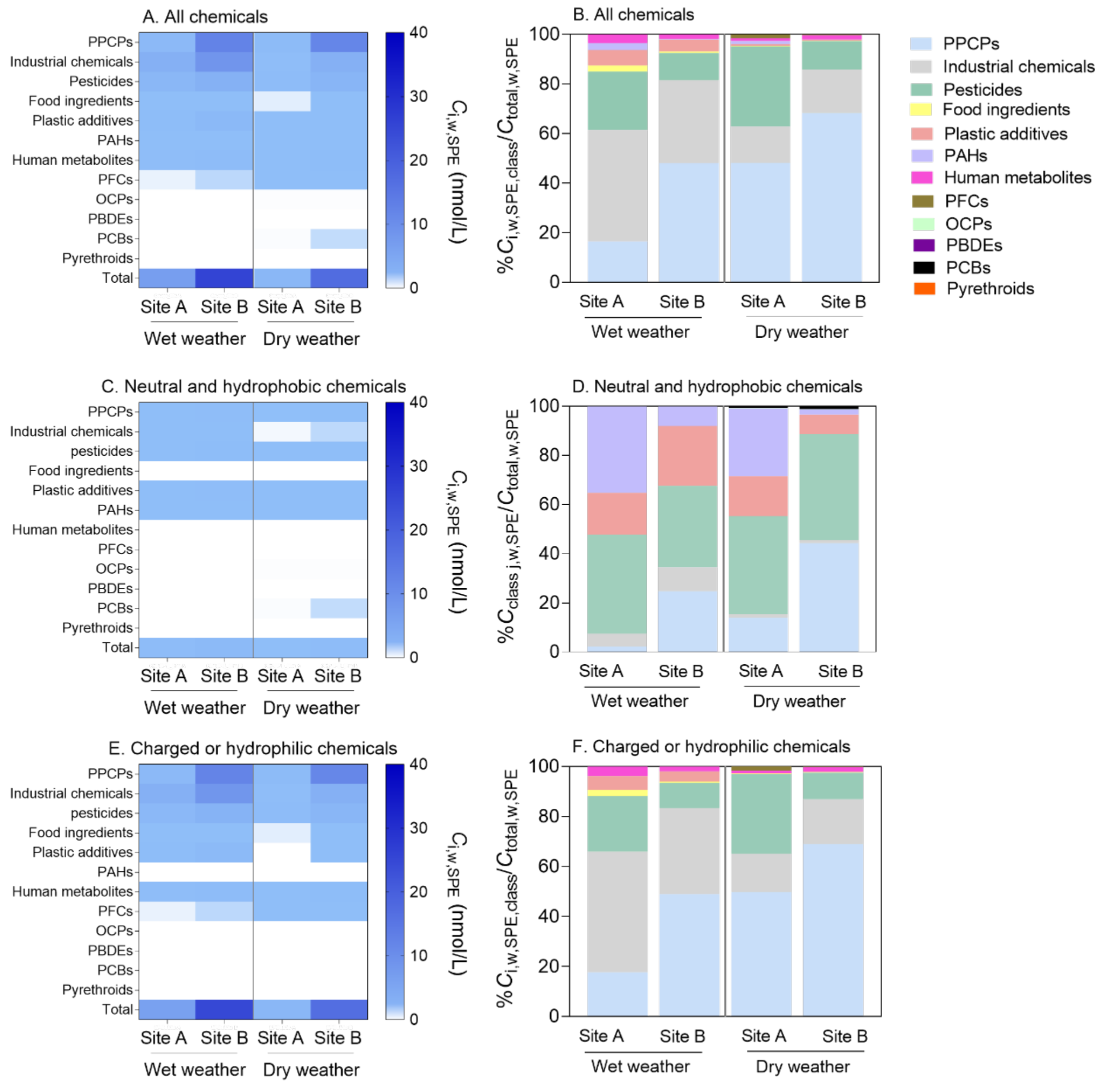

Fig. S2. (A, C and E) Concentrations and (B, D and F) compositions of different chemical groups in the water phase ($C_{i,w,SPE}$) under wet and dry weather conditions. PPCPs: pharmaceuticals and personal care products; PFCs: perfluorinated compounds; PAHs: polycyclic aromatic hydrocarbons; PCBs: polychlorinated biphenyls; OCPs: organochlorine pesticides; PBDE: polybrominated diphenyl ethers.

Text S2. Detailed discussion on chemical concentrations in suspended particulate matter

The role of PAHs at site A during wet weather indicates that the runoff of particles from the nearby road significantly impacted the pollution loading of the studied river during rain events (Fig. S3A). This hypothesis was further evidenced by the chemical profiles in SPM (Fig. S3B). The contribution of PAHs to all SPM-chemicals was nearly 2 times higher in wet weather (81%) than in dry weather (44%) at Site A. Differently, probably due to the impact of WWTP, PPCPs dominated the SPM-bound chemicals at Site B (48% *vs.* 13% in wet and dry weather). Most of these PPCPs were neutral and hydrophobic chemicals (Fig. S3D). It is worth noting that even though industrial chemicals dominated the charged or hydrophilic chemicals, the contribution of plastic additives significantly increased to 18–22% of the total concentrations of charged or hydrophilic chemicals during the rain event at the two sites (Fig. S3F).

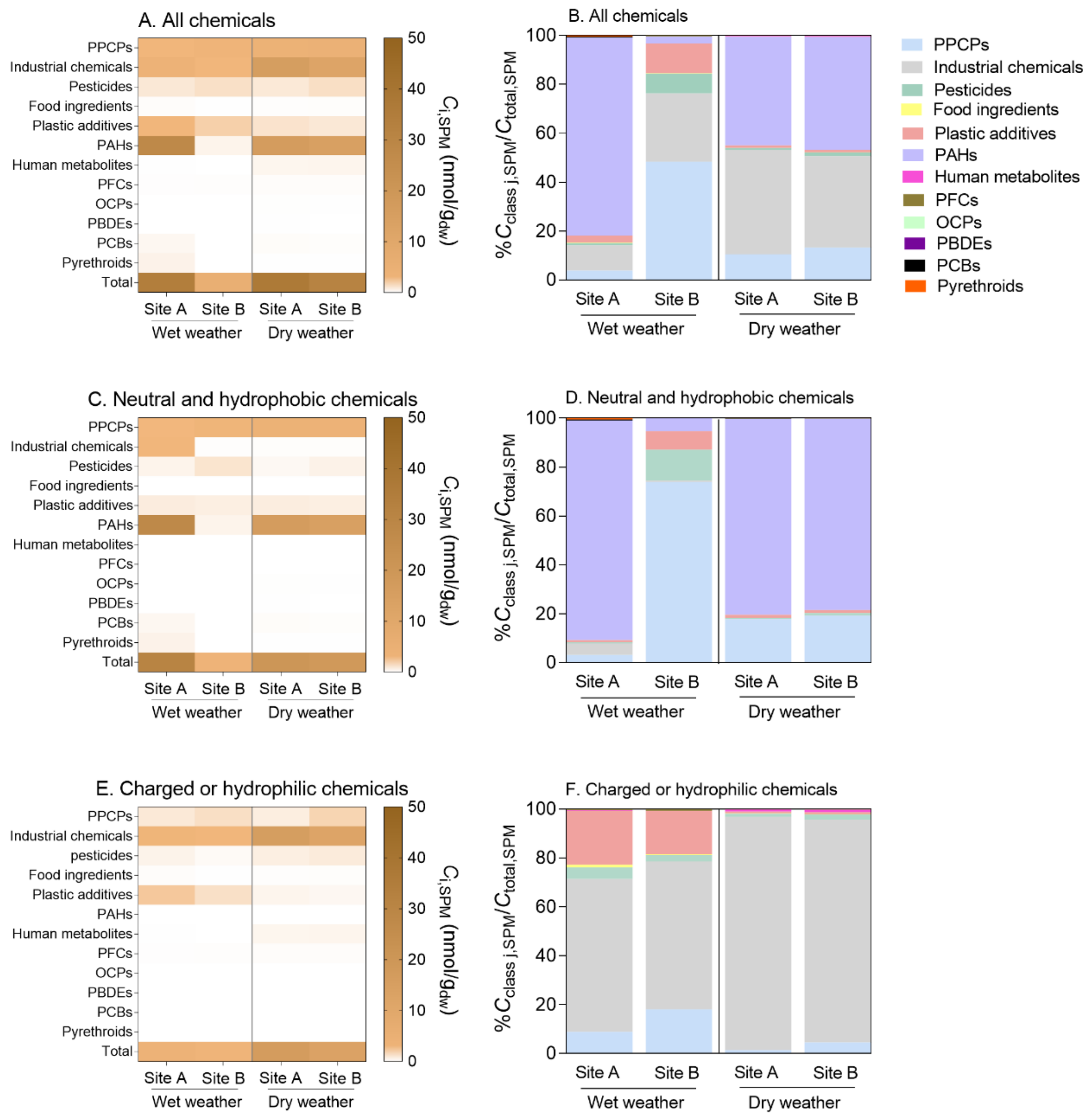

Fig. S3. (A, C and E) Concentrations and (B, D and F) compositions of different chemical groups in suspended particulate matter (SPM) of water ($C_{i,SPM}$). PPCPs: pharmaceuticals and personal care products; PFCs: perfluorinated compounds; PAHs: polycyclic aromatic hydrocarbons; PCBs: polychlorinated biphenyls; OCPs: organochlorine pesticides; PBDE: polybrominated diphenyl ethers.

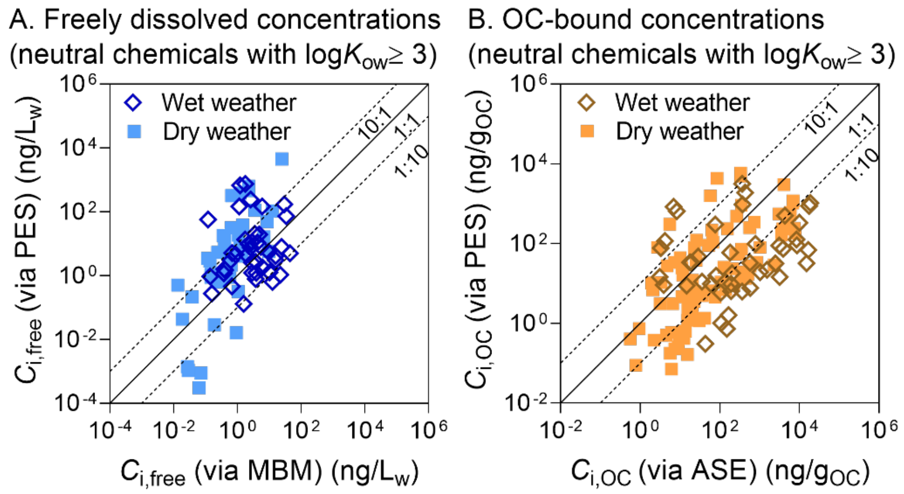

Fig. S4 Figure analogous to Figure 2 but for the neutral and hydrophobic chemicals. (A) Comparisons of freely dissolved concentrations (ng/L) derived with the mass balance model from measured concentration after solid phase extraction ($C_{i,free,SPE}$) and passive equilibrium sampling ($C_{i,free,PES}$); and (B) comparisons of organic carbon (OC)-bound chemical concentrations (ng/g_{OC}) measured by accelerated solvent extraction ($C_{i,OC,ASE}$) and PES ($C_{i,OC,PES}$).

Text S3. Cytotoxicity of chemical mixtures in the water, suspended particulate matter (SPM), and organic carbon (OC)-bound fraction during wet and dry weather using *in vitro* bioassays.

The toxic units (TUs), which refer to the cytotoxicity of chemical mixtures extracted from water, SPM, and the organic carbon (OC)-bound fraction in the three tested *in vitro* bioassays are shown in Fig. S5. The TU_{bio} of water samples were in the range of 0.020−0.033 during the rain event and 0.001−0.032 during dry weather. Unlike the higher chemical concentrations and higher B[a]P-EQ and dichlorvos-EQ values observed during wet weather, the cytotoxicity of water samples collected from the two events displayed no significant differences. This indicates that the cytotoxicity of water was not changed too much by rainfall. Less than 1% cytotoxicity in water was explained by detected chemicals (Table S6 and Fig. S5), which is much less than for the specific endpoints. All chemicals contribute to cytotoxicity, hence this observation is not unexpected.

Most of the SPM samples and their OC-bound fraction showed no cytotoxicity in PPARγ GeneBlAzer and AREc32 bioassays up to the maximum REF of 1.2 g_{SPM}/mL and 0.15 g_{PDMS}/mL. In AhR CALUX, even though no cytotoxicity was observed for SPM at Site A in the rain event, their OC-bound fraction showed approximately 2−4 times lower cytotoxicity than those in dry weather. This is in contrast to the effect data that higher or comparable activation of AhR were observed in PDMS samples after rain events, indicating cytotoxicity decreased but specific effects increased during rainfall. It is worth to note that the cytotoxicity of bulk SPM at Site B decreased by 20 folds after the rain event, whereas that of the OC-bound extract increased by 2.5 folds. More bioavailable cytotoxicity stimulated by rainfall might be attributable to the overflow from the nearby WWTP and should be paid attention. The explained cytotoxicity ranged from non-detected to 0.0008, with higher percentages in OC-bound extracts than bulk samples (Table S6 and Fig. S6).

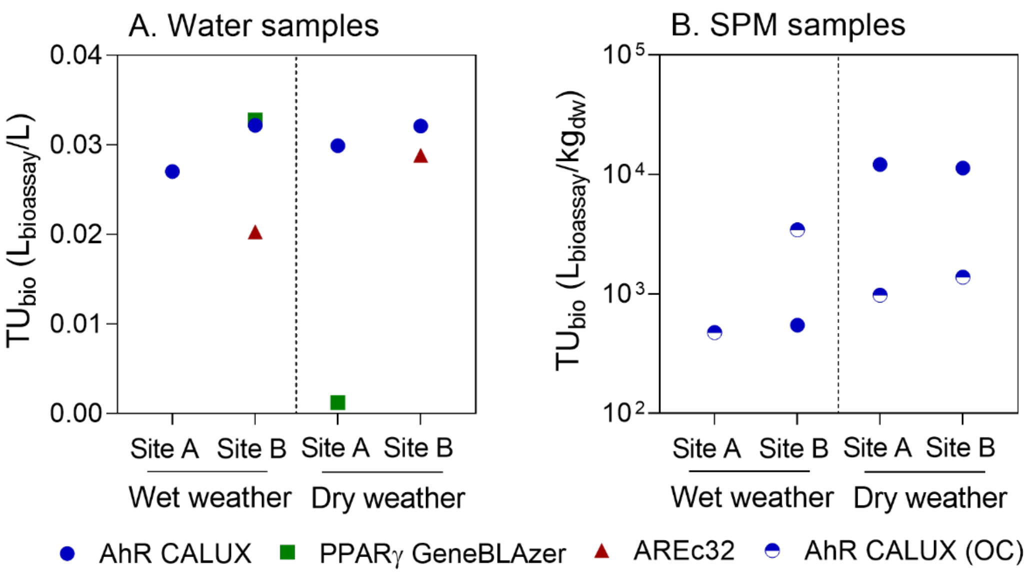

Fig. S5. Toxic units regarding cytotoxicity (TU_{bio}) of chemical mixtures in the (A) water, (B) suspended particulate matter (SPM) and organic carbon (OC) fractions.

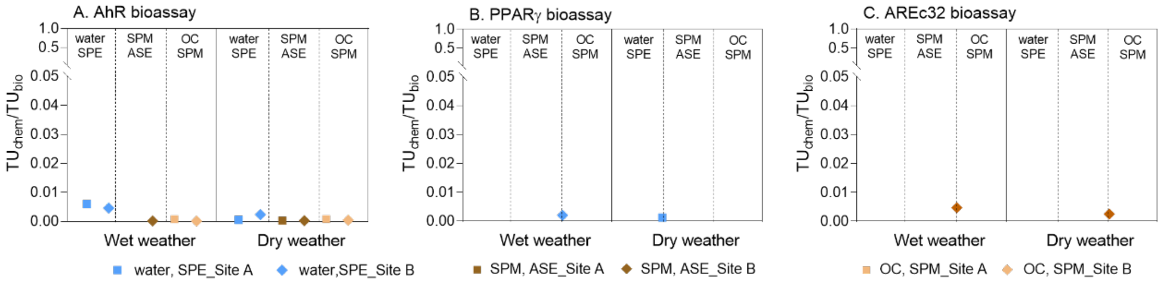

Fig. S6. Contribution of detected chemicals to the observed cytotoxicity using (A) AhR-CALUX cells, (B) PPARγ-GeneBLAzer cells and (C) AREc32 cells.

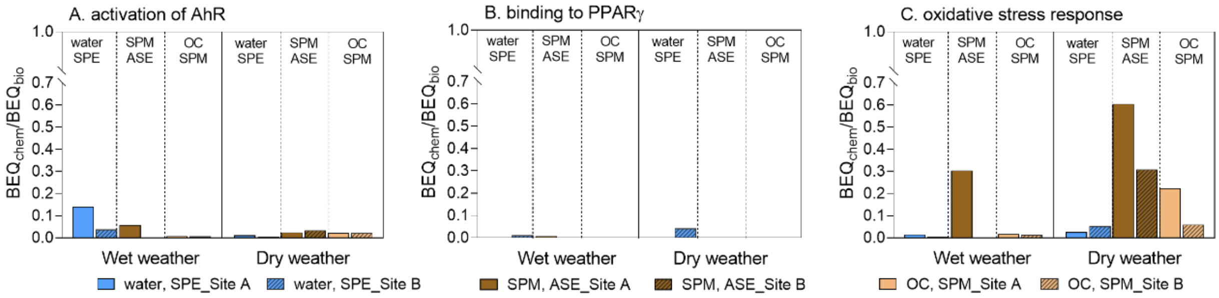

Fig. S7. Contribution of detected chemicals to the observed effects regarding the (A) activation of aryl hydrocarbon receptor activity (AhR), (B) binding to peroxisome proliferator-activated receptor gamma (PPARγ) and (C) oxidative stress response (ratio of bioanalytical equivalent concentration estimated from chemical analysis BEQ_{chem} to those from bioanalysis BEQ_{bio}).

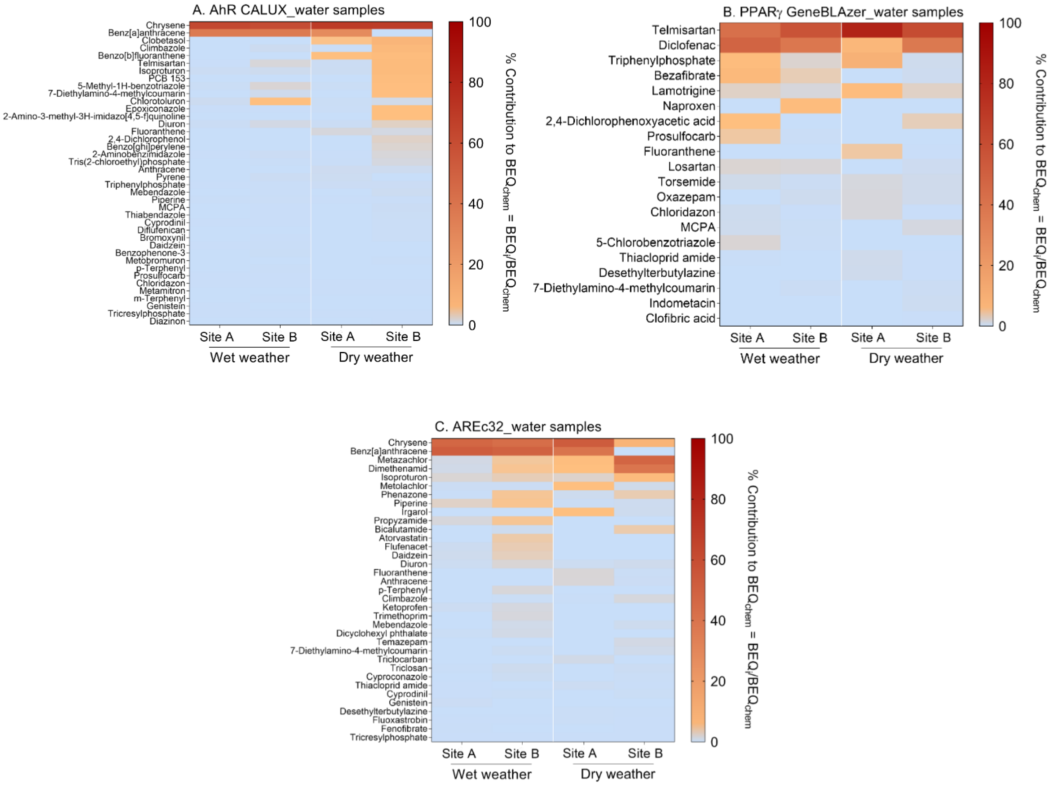

Fig. S8. Contribution of individual chemicals to the total bioanalytical equivalent concentrations (BEQ_{chem}) of water samples for (A) activation of aryl hydrocarbon receptor activity (AhR), (B) binding to peroxisome proliferator-activated receptor gamma (PPARγ) and (C) oxidative stress response during wet and dry weather.

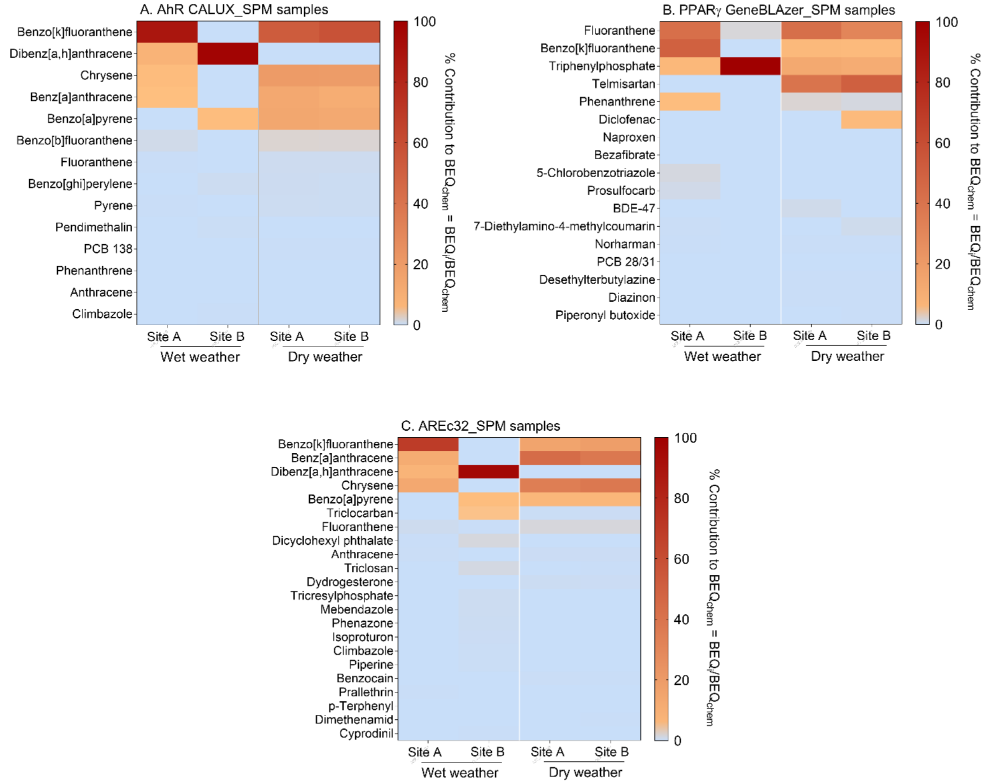

Fig. S9. Contribution of individual chemicals to the total bioanalytical equivalent concentrations (BEQ_{chem}) of bulk suspended particulate matter (SPM) for (A) activation of aryl hydrocarbon receptor activity (AhR), (B) binding to peroxisome proliferator-activated receptor gamma (PPARγ) and(C) oxidative stress response during wet and dry weather.
